# Physiological and pathological skeletal muscle T1 changes quantified using a fast inversion-recovery radial NMR imaging sequence

**DOI:** 10.1038/s41598-019-43398-x

**Published:** 2019-05-02

**Authors:** Benjamin Marty, Pierre G. Carlier

**Affiliations:** 1 Institute of Myology, Neuromuscular Investigation Center, NMR Laboratory, Paris, France; 2 CEA, DRF, IBFJ, MIRCen, NMR Laboratory, Paris, France

**Keywords:** Biomarkers, Neuromuscular disease

## Abstract

We investigated the response of skeletal muscle global T1 under different physiological and pathological conditions using an inversion-recovery radial T1 mapping sequence. Thirty five healthy volunteers, seven patients with Becker muscular dystrophy (BMD) and seven patients with sporadic inclusion body myositis (IBM) were investigated in order to evaluate the effects of gender, age, muscle group, exercise and pathological processes on global T1 values. In addition, the intramuscular fat content was measured using 3-point Dixon and the global T2 and water T2 (T2_H2O_) were determined with a multi-spin-echo sequence. In the muscles of healthy volunteers, there was no impact of age on global T1. However, we measured a significant effect of sex and muscle group. After exercise, a significant 7.7% increase of global T1 was measured in the recruited muscles, and global T1 variations were highly correlated to T2_H2O_ variations (R = 0.91). In pathologies, global T1 values were reduced in fat infiltrated muscles. When fat fraction was taken into account, global T1 values were higher in IBM patients compared to BMD. Global T1 variations are a sensitive indicator of tissue changes in skeletal muscle related to several physiological and pathological events.

## Introduction

Most neuromuscular disorders (NMDs) are accompanied by pathophysiological and structural changes that nuclear magnetic resonance (NMR) imaging can detect. For instance, muscle atrophy, intramuscular fatty infiltrations or inflammation are clearly visible on standard T1 weighted or short tau inversion recovery (STIR) T2 weighted NMR sequences.

More recently, quantitative NMR imaging has emerged as a valuable modality for the atraumatic longitudinal evaluation of these processes during disease progression or therapy monitoring^1^. While FF mapping is commonly used to quantify the late-stage chronic fatty degenerations within skeletal muscles^2^, water T2 (T2_H2O_) maps are generally employed to evaluate disease activity, which refers to non-specific events like inflammatory infiltration, myocyte swelling, sarcoplasmic leakiness or cell necrosis^3,4^. Elevated muscle T2_H2O_ values have been measured in several NMDs such as Duchenne muscular dystrophy (DMD)^5–7^, inflammatory myopathies^4,8–10^ and Pompe disease^11^. This biomarker has been shown to be sensitive to the therapeutic effects of corticosteroid treatment in juvenile dermatomyositis^4^ and in DMD patients^12^.

In the last decade, fast T1 mapping sequences have been developed for cardiac applications and are now routinely employed to characterize the tissue properties of the myocardium. Increased T1 values were measured in acute myocardial infarction, when oedema-like processes are prominent, and in chronic myocardial infarction, when the interstitial volume is increased in parallel with the cell depletion in a fibrotic scar^13^.

In skeletal muscles, while it is well established that global T1 values are strongly decreased at late stages of diseases, when muscle tissues are replaced by fat^14–16^, the effects of water compartmentation and distribution on muscle T1 are still poorly referenced and understood. T1 values were measured after fat suppression, at rest, in the thighs of healthy subjects and no variations were observed between the different muscle groups^17^. The effect of a voluntary exercise on muscle T1 was evaluated in healthy controls^18–20^ and a large T1 increase lasting for several dozens of minutes after the end of the protocol was observed in all studies. This was attributed to an increased perfusion after exercise, but as it also occurred in the presence of vascular occlusion^18^, other mechanisms such as cellular swelling, temperature increase, variations in muscle haemoglobin saturation and intracellular acidification were mentioned to explain these variations. Concerning pathological features, a 13% T1 increase was measured after intramuscular injection of λ-carrageenan in the vastus lateralis of C57BL/j6 mice, a rodent model of local muscle inflammation^21^. In young DMD patients, Matsumora *et al*. measured higher T1 values than in normal subjects, corresponding to the inflammatory response occurring in the early phase of muscle degeneration and regeneration processes^22^. More recently, elevated T1 values were also observed in the canine DMD model^23^.

The goal of our study was to further investigate the variations of skeletal muscle global T1 values in the lower limbs under different physiological and pathological conditions using a recently–proposed inversion-recovery (IR) radial sequence for fast T1 mapping^14^. First, we evaluated the effect of muscle group, gender and age on global T1 in the thighs of healthy volunteers. Then, the variations of global T1 and T2_H2O_ were compared after a plantar flexion exercise in another group of healthy subjects. Finally, the extent of global T1 changes occurring during pathological processes was evaluated in two different diseases: sporadic inclusion body myositis (IBM) and Becker muscular dystrophy (BMD). Sporadic IBM is an idiopathic inflammatory myopathy characterized by the combination of inflammatory and myodegenerative features with multi-protein aggregates in muscle tissues^24^, while BMD is a dystrophinopathy presenting a progressive replacement of the muscle tissues by fat which is accompanied by a less severe inflammatory response^25,26^.

## Results

### Healthy volunteers at rest

**Figure 1:**
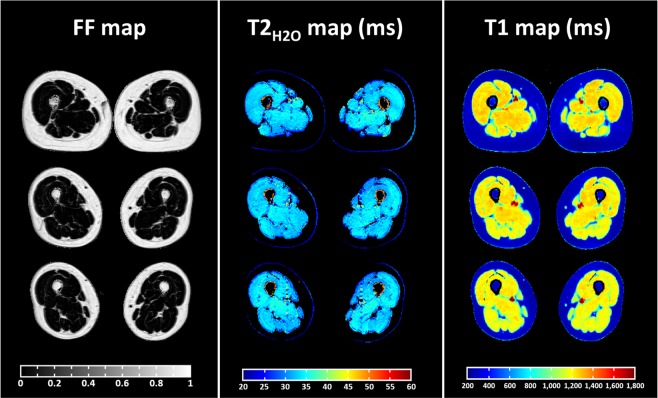
Representative fat fraction (FF), water T2 (T2_H2O_) and T1 parametric maps obtained on a 51 y.o. healthy woman at different slice levels in the thighs. As expected on healthy subjects, the intramuscular fat content was low with FF below 0.05 and homogeneous T2_H2O_ values ranging from 33 to 35 ms were observed in the different muscle groups. On the T1 maps, the high contrast to noise ratio, combined with the millimetre spatial resolution allowed to differentiate several structures: subcutaneous fat, bone marrow, large blood vessels, several muscle fasciae and skeletal muscles wherein T1 ranged between 1,200 and 1,250 ms.

Global T1, T2_H2O_, and FF maps are shown at different slice levels in the thighs of one healthy volunteer (Fig. 1). The mean values measured in the quadriceps and hamstrings muscles of all subjects are summarized in Table 1. The effects of muscle group, gender and age on the three variables were assessed using a repeated-measures analysis of covariance (ANCOVA). There was a significant effect of muscle group (p < 0.001) and gender (p = 0.02) on global T1 values, but no significant effect of age (p = 0.479). The muscle × gender and muscle × age interactions were not significant (p = 0.430 and 0.907, respectively). The ANCOVA revealed no significant effect of muscle group and sex on T2_H2O_ values (p = 0.115 and p = 0.054, respectively), but a significant effect of age (p = 0.005). There were no significant interactions between muscle group and age, nor between muscle group and gender for this variable (p = 0.230 and p = 0.629, respectively). As for T2_H2O_, there was no significant effect of muscle group and gender on FF values (p = 0.98 and p = 0.231, respectively), but a significant effect of age was observed (p = 0.007). The muscle group × age interaction was also significant (p = 0.001), but not the muscle group × gender interaction (p = 0.289).

**Table 1:** Compartmental fat fraction (FF), water T2 (T2_H2O_) and T1 values measured in the different muscle groups of healthy subjects.

Muscle group	Men			Women		
	FF	T2_H2O_	T1	FF	T2_H2O_	T1
Quadriceps	0.025 ± 0.012	32.9 ± 0.8	1221 ± 21	0.028 ± 0.010	33.4 ± 0.8	1242 ± 21*
Hamstrings	0.032 ± 0.020	32.7 ± 0.9	1184 ± 32^†^	0.038 ± 0.015	33.4 ± 1.0	1212 ± 29^†,^*

Symbols † and * depicts variables showing significant differences among muscle group and gender, respectively, according to the repeated measures analysis of covariance with Bonferroni post-hoc-test (p < 0.05).

### Healthy volunteers after concentric exercise

**Figure 2:**
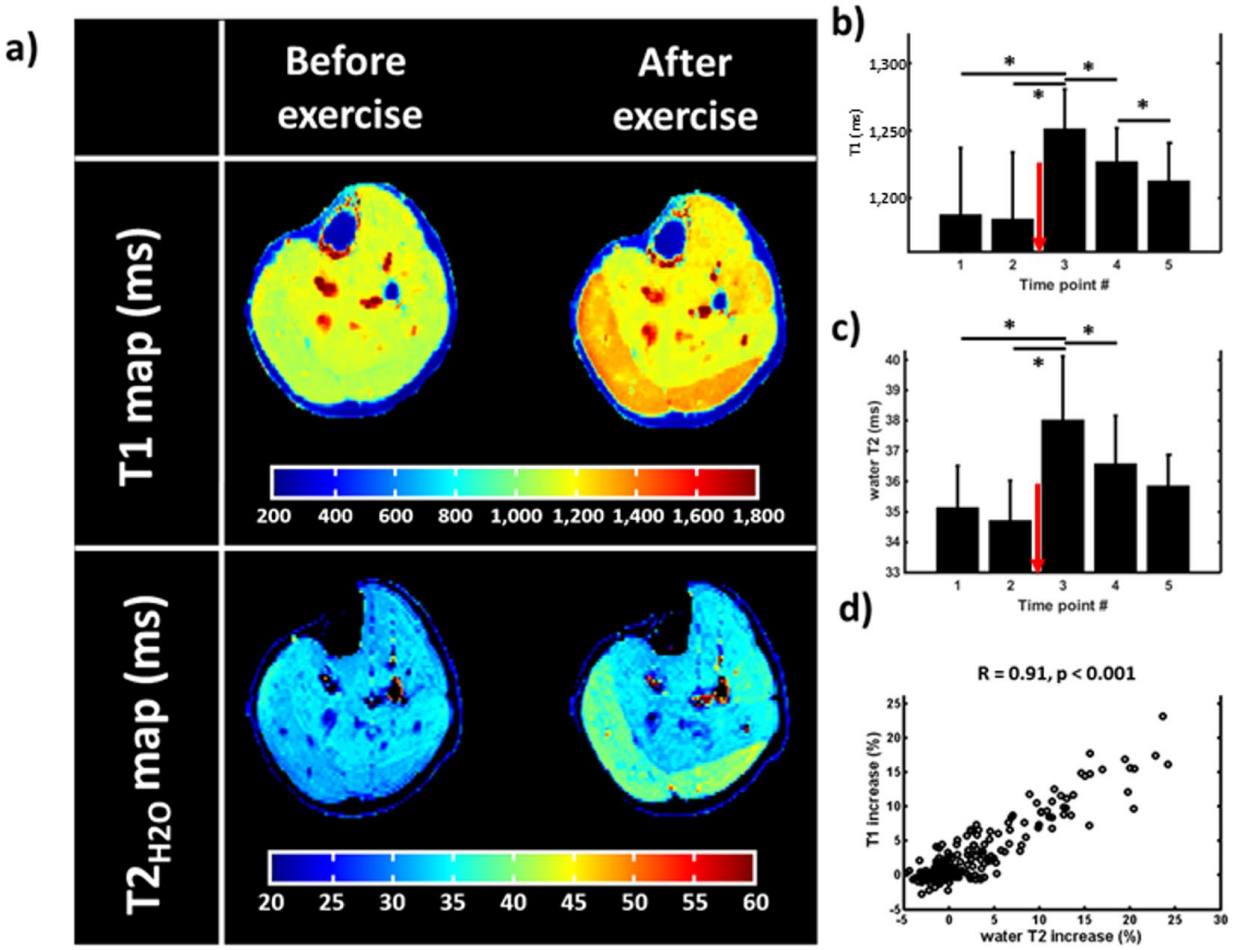
(**a**) T1 maps acquired on the right leg of a 29 y.o. healthy man before and immediately after an 8-minutes concentric plantar flexion bout of the right foot. (**b**,**c**) Temporal evolution of the T1 measured on the right gastrocnemius lateralis and medialis during the entire exercise protocol. The exercise bout was performed between time points 2 and 3 (red arrow). Repeated measures ANOVA tests revealed significant variations of T1 between the 5 acquisition points (*Bonferroni post-hoc tests, p < 0.05). (**d**) Water T2 (T2_H2O_) and T1 increases were measured between time points 2 and 3 for all volunteers, and a linear correlation was observed between the two variables during this protocol (Pearson correlation coefficient R = 0.91).

Global T1 and T2_H2O_ maps acquired before and after a plantar flexion bout demonstrate a large increase of both relaxation times post-exercise, mainly in the gastrocnemius medialis (GM) and gastrocnemius lateralis (GL) muscles (Fig. 2a). Repeated-measures analysis of variance (ANOVA) with Bonferroni post-hoc test revealed a significant increase of T1 and T2_H2O_ after exercise on the entire cohort of subjects (7.7% and 8.5%, respectively) in the triceps surae group, which was followed by a progressive return to baseline values (Fig. 2b,c). T1 and T2_H2O_ variations measured at the different time-points after plantar flexion were also highly correlated (R = 0.91, p < 0.001, Fig. 2d).

**Figure 3:**
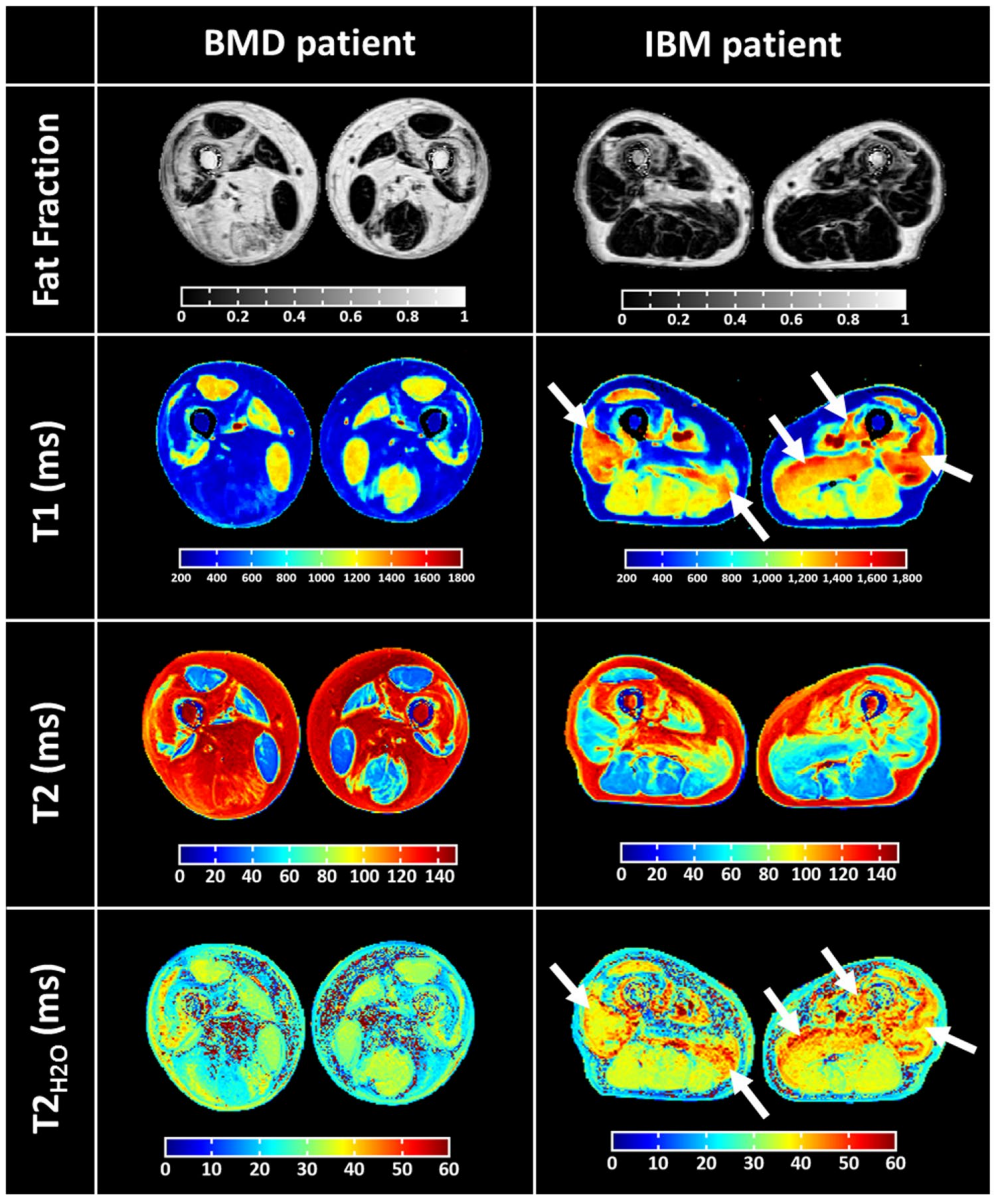
Representative fat fraction, global T1, global T2 and T2_H2O_ parametric maps obtained in the thighs of a 64 y.o. man with Becker muscular dystrophy (BMD) and a 76 y.o. man suffering from inclusion body myositis (IBM). Areas of increased global T1 and T2_H2O_ are depicted by white arrows.

### NMD patients

In patients with NMDs, fat infiltrations were present in several muscle groups and corresponded to areas of reduced global T1, and increased global T2 values (Fig. 3). However, in several IBM patients, regions of elevated global T1 could be detected, and were easily observed in muscle groups with low fat content. Interestingly, these muscle groups were also characterized by increased T2_H2O_.

First, relaxation parameters were measured in ROIs with low fat content (<0.1) and are summarized in Table 2. In these regions, FF were neither significantly different between IBM and BMD subjects nor between muscle groups. Water T2 was significantly higher in the quadriceps and the hamstrings of IBM subjects compared to BMD. In the IBM patients, T2_H2O_ were significantly higher in the quadriceps compared to the hamstrings. On the other hand, global T1 was only significantly higher in the quadriceps of IBM subjects compared to BMD. For both pathologies, as observed in healthy volunteers, global T1 was significantly higher in the quadriceps than in the hamstrings.

**Table 2:** Compartmental fat fraction (FF), water T2 (T2_H2O_) and T1 values measured in the muscle groups of NMD subjects where FF < 0.1.

Muscle group	BMD			IBM		
	FF	T2_H2O_	T1	FF	T2_H2O_	T1
Quadriceps	0.056 ± 0.008	32.8 ± 0.9	1195 ± 32	0.059 ± 0.021	36.9 ± 2.1*	1264 ± 55*
Hamstrings	0.060 ± 0.016	32.1 ± 1.1	1137 ± 11^†^	0.063 ± 0.021	35.0 ± 1.5^†,^*	1159 ± 55^†^

Symbols † and * depicts variables showing significant differences among muscle group and pathology, respectively, according to the repeated measures analysis of covariance with Bonferroni post-hoc-test (p < 0.05).

**Figure 4:**
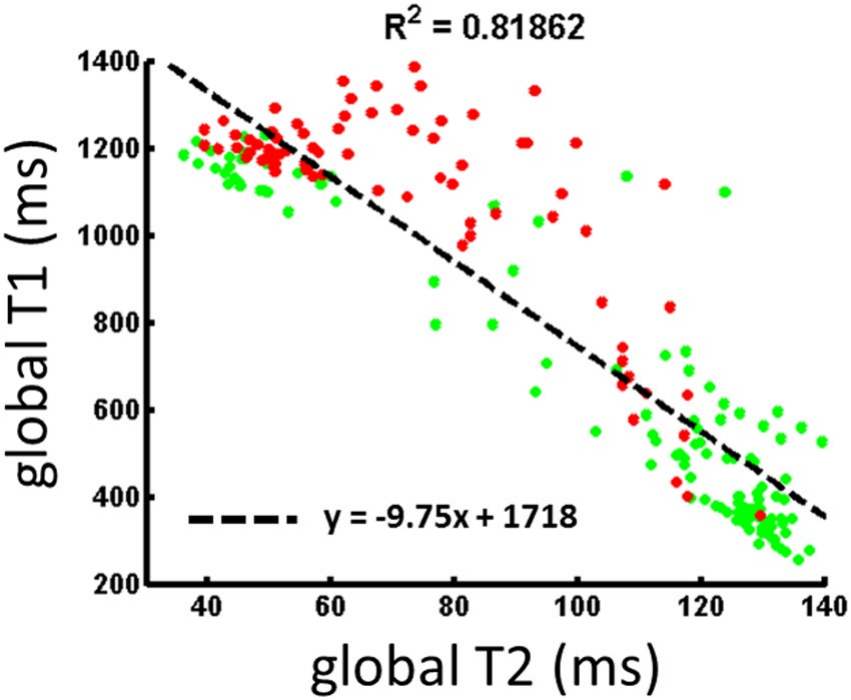
Comparison between global T1 and global T2 in the muscles of NMD patients, Green points: normal T2_H2O_ (<34.4 ms), Red points: high T2_H2O_ (≥34.4 ms).

To go further, all muscles were included in the analysis. First, the ROIs of the two patient groups were pooled and we found a significant correlation between global T1 and global T2 values (Fig. 4, R^2^ = 0.81, p < 0.01). However, this plot clearly depicts that some points, which are located above the regression curve do not follow the same linear relationship between global T1 and global T2. A colour code was applied to the experimental data-points based on the normative T2_H2O_ values measured on healthy men (T2_H20,norm_ = 32.8 ± 0.8 ms): green points corresponds to muscles with normal T2_H2O_ (T2_H2O_ <T2_H20,norm_ +2 SD = 34.4 ms) and red points correspond to regions with high T2_H2O_ (T2_H2O_ ≥34.4 ms). Most of the points located above the regression curve are characterized by high T2_H2O_ values.

**Figure 5:**
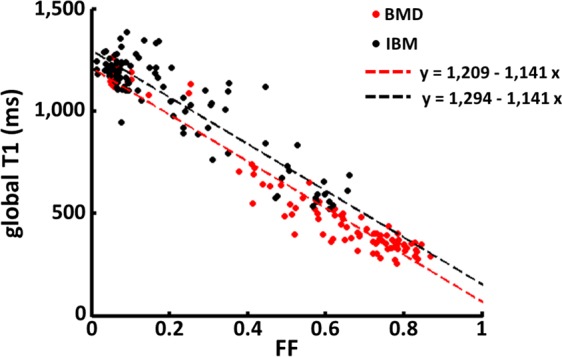
Global T1 values in the muscles of the two patients groups were plotted as a function of FF (Becker muscular dystrophy (BMD) in red and inclusion body myositis (IBM) in black). A multiple linear regression was calculated to predict T1 based on FF and pathology and a significant regression was found (F = 1,581, p < 0.001), with an R^2^ of 0.942 (dotted lines).

Then, T1 values in the individual muscles were plotted as a function of FF for the two populations of patients (Fig. 5). A multiple linear regression was calculated to predict global T1 based on FF and pathology. A significant regression was found (F = 1,581, p < 0.001), with an R^2^ of 0.942. Subject’s predicted T1 was equal to 1,124–1,141 × (FF) + 85 × (pathology), with pathology coded as 1 = BMD and 2 = IBM. For an equivalent fat content, there was a significant increase of T1 in IBM patients of 85 ms. Both FF and pathology were significant predictors of global T1 (both p < 0.01). Because the two groups were not exactly matched in age, this parameter was also included in the regression. The F-test revealed that there was no significant improvement of the regression when age was taken into account (F = 0.07, p = 0.791), and that there was no collinearity between age and pathology to predict T1 variations.

Finally, we also observed a significantly higher T2_H2O_ value, averaged across all muscle groups, in IBM patients compared to BMD subjects (mean T2_H2O_ = 35.1 ms vs 30.4 ms, p < 0.01).

## Discussion

In this study, we investigated skeletal muscle tissues T1 variations under different physiological and pathophysiological conditions using a fast IR-radial T1 mapping sequence. We found that global T1 changes related to muscle tissues structural changes represents an additional sensitive information regarding the study of NMDs or basic physiology, that could be complementary to more conventional variables such as FF and T2_H2O_.

Technical bolts related to the long acquisition time of gold standard T1 mapping sequences are now overcome, and this fundamental NMR parameter may be evaluated in fast protocols compatible with clinical research. Here, the short acquisition time of the IR-radial sequence represents a valuable advantage of the T1 approach when fast imaging is mandatory. Quantitative imaging might become feasible in young children, or severely affected patients, who cannot remain motionless in the scanner for long periods of time. In our different protocols, scanning was well tolerated by all subjects, from young healthy volunteers down to 7 years of age to older patients up to 79 years of age.

In the lower limbs of healthy volunteers, we observed significant variations of global T1 that can be attributed to different physiological conditions. First, women skeletal muscles exhibited higher global T1 values than men that could not be explained by differences in the intramuscular fat content. This could instead result from a difference of the mean haemoglobin level and resting blood flow between men and women^27,28^. A gender dependence of T1 values has already been measured in the myocardium of healthy volunteers and disappeared after normalization to blood T1^29^. Independently of gender, global T1 values were also significantly higher in the quadriceps muscles than in the hamstrings. This might reflect differences in fibre type composition in the different muscle groups, type I fiber being associated to a slightly denser capillary network than type II fibers^30^.

Although a significant effect of age was observed in healthy volunteers for FF and T2_H2O_, this variable had no impact on global T1 in any muscle group. The results for FF and T2_H2O_ are in line with previous studies regarding the effect of aging on these standard NMR biomarkers. Qualitative^31,32^ as well as quantitative^33^ NMR imaging sequences have already demonstrated the significant increase in the amount of intramuscular fat with age. Higher T2_H2O_ values were as well reported in the legs of old animal models^34–36^ and in the thighs of elderly volunteers compared to young adults^33^. These T2_H2O_ changes were again attributed to the shift in muscle fibre composition with age, based on several studies showing that type II fibres content was decreasing with age^37–40^. The lack of correlation between age and global T1 arises from the inability of the proposed NMR sequence to separate between water and fat signal contributions. While an increased fat content would have resulted in lower muscle T1 values, this effect was probably thwarted by an increase in water T1 due to the shift in fibre types.

Quantitative T1 and T2_H2O_ maps were also acquired on healthy volunteers before and after voluntary contractions of the right leg. The effect of exercise on relaxation times was in accordance with previous reports where increased values were measured in the most recruited muscles groups for several minutes after the end of the effort^18–20^. To meet the important metabolic demand during exercise, the intravascular volume expends and is associated to a short term increase of both T1 and T2_H2O_ relaxation times. However, comprehensive studies of muscle T2_H2O_ changes in exercising muscles revealed that the predominant contribution was intracellular and resulted from an accumulation of the end-products of the anaerobic metabolism within muscle cells, causing a decrease of pH and an increase of intracellular volume^41,42^. As our results showed an excellent correlation between T1 and T2_H2O_ variations during the recovery period after exercise, these mechanisms are certainly the main cause of the global T1 increase following exercise stimulation.

Regarding the two NMDs considered in our study, we noticed that several pathological processes were modulating the muscle longitudinal relaxation time. As expected, for both BMD and IBM patients, global T1 was largely decreased when the intramuscular fat content was increased. Beyond that effect, we also demonstrated that the inflammation process occurring in IBM patients in parallel with the chronic fatty infiltration could be detected on T1 maps and resulted in increased values compared to the BMD group. Increased T2_H2O_ values in patients with sporadic IBM have already been reported using either qualitative imaging approaches such as T2 weighted STIR imaging^43,44^ or quantitative water T2 mapping sequences^10,45^ and this feature has been attributed to oedema-like changes reflecting inflammation processes. To our knowledge, however, this is the first time that these pathological features may also be related to increased T1 values in patients with inflammatory myopathies.

The main limitation of the T1 mapping sequence used in our study is that the different contributions of water and fat protons on the global muscle signal are not separated. Inflammation-related processes, resulting in an increase in global T1 may be differentiated from chronic fatty degenerations leading to decreased T1 values if the two processes are not mixed. However, when the two phenomena occur in parallel, as observed in the IBM patients, the inflammatory response would have been hidden by the fatty degenerations if T1 maps had not been analysed along with the corresponding FF maps. In future steps, a multi-component analysis of the T1 recovery should be implemented to clearly separate water and fat proton signals that would allow using water T1 as a standalone biomarker. Very recently, fast sequences based on the MR fingerprinting principle^46^ have been proposed to simultaneously derive water T1 and FF in fatty infiltrated regions^47–49^. With their fast acquisition time (around 10–20 seconds per slice), they represent a more robust basis to evaluate the potential role of water T1 quantification as a biomarker of skeletal muscle structural and functional alterations in NMDs. Moreover, because T2_H2O_ maps are generally computed from multi-spin echo sequences that are limited by high specific absorption rate and long acquisition time, water T1 quantification might become a concurrent to the T2_H2O_ approach for monitoring disease activity in NMDs.

In conclusion we demonstrated that muscle T1 variations are a sensitive indicator of structural changes in skeletal tissues related to several physiological and pathological events. Further studies should evaluate the potential role of T1 mapping as a biomarker for monitoring NMDs.

## Experimental

### Study population

In this study, thirty-five healthy volunteers were included. Twenty-five of them had their thighs scanned at rest (13 men, 36.2 ± 24.6 y.o and 12 women, 37.6 ± 23.2 y.o), while the other ten (7 men, 35.0 ± 8.6 y.o and 3 women, 30.3 ± 4.6 y.o) were imaged in the right calf before and after performing an 8 minutes plantar flexion bout. This exercise protocol was realized inside the scanner using a custom-built non-magnetic ergometer. Two baseline measures were acquired before exercise, and three measures were obtained immediately after the end of the procedure with a 4 minutes temporal resolution. Seven patients with BMD (7 males, 60.9 ± 5.8 y.o.), and seven patients with IBM (7 males, 69.1 ± 9.9 y.o.) were also investigated at rest at the thighs level. The protocol was approved by the local ethics committee (Comité de Protection des Personnes (CPP) Ile de France VI) and written informed consent was obtained from all subjects. All experiments were performed in accordance with the guidelines and regulations of the Declaration of Helsinki.

### NMR imaging protocol

The NMR imaging protocol was carried-out at 3 T (Magnetom Prisma^Fit^, Siemens Healthineers, Erlangen, Germany) with the patients positioned feet first in supine position. For the protocol without exercise, the body coil was used for RF transmission. One 18-channel flexible phase array coils was wrapped around the thighs and used in combination with a 32-channel spine coil for signal reception. For the exercise protocol, a 15-channel volume transmit/receive knee-coil, compatible with the ergometer setup, was positioned around the right calf of the volunteers.

The T1-mapping sequence consisted of a single inversion pulse, followed by the acquisition of a 1000-spokes fast low angle shot (FLASH) radial echo train. Sequence parameters were: echo time (TE)/echo spacing (ES) = 2.75/5.08 ms resulting in an echo train length of 5,080 ms; flip angle (FA) = 8°; receiver bandwidth = 820 Hz/px; a 10 ms hyperbolic secant inversion pulse with a delay time (TD) = 21 ms; field of view = 350 mm^2^; resolution = 320 points/spoke; resulting in a pixel size of 1.1 × 1.1 mm^2^; 5 slices (slice thickness = 10 mm; slice gap = 25 mm), repetition time (TR) = 10 s; acquisition time (T_acq_) = 50 s. The k-space coverage was optimized using a golden angle scheme between 2 successive spokes. Global T1 maps were obtained according to the procedure previously described^14^.

Fat fraction quantification was performed applying the reference 3-point Dixon technique^50^ using a three-dimensional gradient echo sequence with three TEs (TE1/TE2/TE3 = 2.75/3.95/7.55 ms). The other sequence parameters were: TR = 10 ms, spatial resolution = 1 × 1 × 5 mm^3^; FA = 3° and T_acq_ = 1 min 36 s.

An MSME sequence was acquired with the following parameters: TR = 3000 ms; nominal flip angles = 90°/180°; truncated sinc3 excitation and refocusing pulses (2.56 and 3.84 ms, respectively); a train of 17 echoes (TEs from 9.5 to 161 ms; ES = 9.5 ms); FOV, 224 × 448 mm^2^; pixel size, 1.4 × 1.4 mm^2^; bandwidth, 446 Hz/pixel; 11 slices (slice thickness = 10 mm; slice gap = 25 mm); T_acq_ = 3 min 41 s. From the observed signal decays, a single-component and a two-component EPG approaches were used to measure both global T2^51^ and T2_H2O_ values^4^, respectively.

### Regions of interest

For the protocol without exercise, regions of interest (ROIs) were manually drawn, using a free software tool (www.itksnap.org), in the left and the right limbs on the MSME images in the vastus lateralis (VL), the vastus medialis (VM), the vastus intermedius (VI), the rectus femoris (RF), the semimembranosus (SM), the semitendinosus (ST) and the biceps femoris (BF) of the thighs. For the exercise protocol, ROIs were drawn in the right gastrocnemius medialis and gastrocnemius lateralis muscles. The ROIs were delimited to the interior of the muscle avoiding fasciae and large blood vessels.

### Statistical methods

Results are presented as mean ± standard deviation (SD) in the different muscle groups and the statistical analysis was performed using SPSS 22 (IBM, Armonk, NY, USA).

For better readability in the healthy subjects, the different regions of interest were pooled in two muscle groups: quadriceps (comprising RF, VI, VL and VM) and hamstrings (comprising BF, ST and SM). Statistical comparisons were made for global T1, T2_H2O_ and FF values using a repeated-measures ANCOVA, muscle group being considered as a within variable, gender as a between variable and age as a covariate. We considered p < 0.05 to be statistically significant.

The impact of the voluntary exercise on global T1 and T2_H2O_ values was assessed by repeated-measure ANOVA in the triceps surae muscle group, which was the most activated at the end of the plantar flexion bout. A linear regression analysis was performed to determine correlations between T2_H20_ and T1 variations in all muscle groups at the different time-points.

For the NMD patients, as the involvement pattern in the two diseases is known to be highly heterogeneous, the different muscles were analysed independently. A multiple linear regression was performed using global T1 as a dependent variable and FF and pathology as explanatory variables. As the age of the IBM and BMD groups were not exactly matched, this variable was also tested as an explanatory variable in a second step.

## Data Availability

The datasets generated during the current study are available from the corresponding author upon reasonable request.
